# Medical Reminiscences of New York

**Published:** 1860-03

**Authors:** D. C. O’Keefe

**Affiliations:** Atlanta, Ga.


### ARTICLE II.

*Medical Reminiscences of New York.*—By D. C. O'KEEFE, M. D., Atlanta, Ga.

### BELLEVUE HOSPITAL.—SERVICE OF DR. METCALFE.

*Case* 1.—Pneumonia associated with delirium tremens in a man 45 years old; has been in three or four days. His condition two days ago was very unfavorable; pulse 130; had the prune-juice expectoration, which some regard as always indicating an unfavorable result, but which is not correct—he had seen a few cases in which it occurred and the patient recovered.

This man's condition now is much more promising; pulse 80–90. The treatment has been brandy, carbonate of ammonia, nourishing diet, and everything calculated to build him up; nothing of a depressing character has been given, and you see the result. Had he been bled, and purged, and vomited, and mercurialized, he would have been dead now.

This case shows the importance of not treating names—don't allow a local inflammation to divert your attention from the general condition of the constitution. The connection of delirium tremens with these cases of pneumonia is an interesting subject. Some men of intemperate habits have a condition of the system constantly present which might be denominated latent delirium tremens, and which is liable to break out on the application of any exciting cause. Thus, pneumonia, or any acute disease, will develope it; so will an accident, such as a fracture or dislocation.

DEAD HOUSE.

*Case* 2.—Dr. Metcalfe was called, some months ago, to an old gentleman in the city, who was suffering from diabetes. He passed large quantities of urine of specific gravity 1040, and containing sugar in abundance. The liver projected three or four inches below the false ribs, and he distinctly felt the gall-bladder of considerable size below the liver. He was very fat, weighing about 200 pounds; had a voracious appêtite for food and drink, of which he partook in great abundance, as is usual in this disease; drank freely of beer, ale and whiskey. He (Dr. M.) supposed he had fatty liver, so common in persons of such habits. From being so fat, he became reduced almost to a skeleton; had an attack of diarrhœa, which proved very troublesome—would be up sometimes every half hour—it yielded in ten days to tannin and Dover's powder.

Some time ago, he went in the country, and had a stroke of apoplexy, which soon passed off and left no trace behind. Some time after, he had another stroke, of greater severity, and, to see him in this condition, Dr. M. was sent for. He found him puffed or œdematous, instead of being lean and sallow, and still laboring under his fit of apoplexy. He died that night, and a post-mortem was solicited and obtained. On opening the head, a thin layer of blood was found on the superior surface of the left hemisphere; some also on the corpus striatum, and slight congestion of the arachnoid. There was not as much evidence of disease in the brain as he had expected to find.

On opening the thorax, the apex of one lung was found infiltrated with whitish tubercular matter, a specimen of which he presented. The liver was found slightly enlarged, weighing 5 lbs., not near as much as he was led to expect. The kidneys were slightly enlarged—not more than might be expected in organs that had performed a very active function. Above the right kidney, at the locality of the supra-renal capsule, (which could not be found,) was discovered a tumor, of an oblong form, somewhat the shape of an infant's head after a protracted labor, and quite as large. It extended from the upper surface of the kidney to the under surface of the diaphragm, to both of which it was attached by loose cellular tissue. The tumor was composed of two distinct parts united together by a pedicle in the shape of an hour-glass; the pedicle was not distinctly brought to light until cellular tissue which enveloped it, and filled up the constricted portion, was dissected away.

The superior, or diaphragmatic half, on being cut into, presented a solid appearance, and was of an encephaloid character, which he believed to be cancerous. The upper renal half was a multilocular cyst filled with fluid.

The points of interest in this case are numerous: 1st The size of the liver, which he expected to weigh 7 or 8 pounds, weighed but five; its projection four inches below the false ribs, being due to the tumor pressing it down. Enlarged liver, then, may be due to various causes, such as: fatty degeneration, cirrhosis in first stage, carcinoma, chronic engorgements from residence in warm climates and from cardiac affections, collections of fluid in right pleural cavity, and tumors in vicinity pressing down the liver, as in this case. 2d. The co-existence of carcinoma with tubercular deposits in the system, which was regarded a very rare occurrence. The tumor will be subjected to microscopical examination by Dr. Gouley, and the result will be reported at another time.

*Case* 3.—The lungs of a patient who died of pneumonia were presented, exhibiting the stage of red hepatization in one lung and grey hepatization in the other. He believed the books erred in giving three stages of pneumonia, viz: that of congestion, red hepatization and grey hepatization or purulent infiltration. Now, in regard to the two last conditions, he believed them to be quite different states. The former, or grey hepatization, was a condition through which every inflamed lung passed, on its way to convalescence; the latter, or purulent infiltration, was a period in the disease in which the patient was nearly gone. He did not believe there were but two stages of the disease. The grey hepatized lung presented the fine granular appearance, hardly perceptible to the naked eye, except in a clear light, of miliary tubercular infiltration, which he believed sometimes occurred in one day.

DR. MARCOE'S CLINIQUE.

*Case* 4.—Eczema of the face in a child three months old. Consists of fine accuminated vesicles, filled with thin fluid, which burst and discharge their contents, and form thin whitish scaly scabs. It is consistent with good health, and hard to cure. *Treatment*—Oxide of zinc and weak stramonium ointment.

*Case* 5.—Torticollis in young woman, aged 20 years. Consists in physical shortening of the sterno-cleido-mastoid muscle; no other change in muscular fibres. This case was noticed the first week after birth, and gradually got worse until it reached its present condition. There are two forms —that which comes on gradually, and that which occurs suddenly. The treatment is mechanical—two modes of: 1st. Forcible extension with hands in direction opposite to that which the deformity assumes, until the fibres are heard to crack, and the curvature has been overcome. This is Dr. Buck's method, and has been successful in his hands. The patient must be put under the influence of anæsthetics, and the head kept straight by mechanical appliances. The second mode of treatment consists of a sub-cutaneous section of the muscle, near its sterno-clavicular attachment, and then maintaining the head straight by mechanical appliances, as in the other plan.

*Case* 6.—Psoas abscess of two months standing in a boy, 7 years old. There is emaciation and loss of appetite; stiffness in affected side; cannot bend down to pick up an object from the floor without pain. It is hard to distinguish from Pott's disease of the vertebræ—is often associated with it, and therefore of scrofulous origin. In the latter case, the prognosis is unfavorable, the discharge being kept up by the diseased condition of the vertebræ. The pus takes the course of the psoas muscle; gets under Poupart's ligament, and finds exit in front of upper third of thigh; the pus discharged has a *curdy* appearance, which is characteristic. Prognosis sometimes favorable, often not so. *Treatment*—Hygienic, dietetic and tonic. A diagnostic test of this disease is an inability to bury the tips of the fingers in the iliac fossa close to the ilium, as in health. This will lead to the suspicion that there is some disease present which fills up the iliac fossa.

*Case* 7.—Iliac abscess of eighteen months standing, in a man 45 years old, supposed to be caused by a sprain received about the sacro-iliac articulation. It exists somewhat in same locality as psoas abscess, but differs from it in not being constitutional—not connected with diseased vertebræ—not scrofulous, and does not have the unhealthy curdy pus. Prognosis generally favorable. *Treatment*—Open when indicated.

*Case* 8.—Woman with inflamed fore-arm presented at last clinique. It is a case resulting from pus burrowing in the theca of tendons. Whitlow is an example.

These are cases where early surgical interference does a vast amount of good. As soon as the deep-seated inflammation is discovered, lay the part open deeply and freely. It will prevent suppuration, the destruction of periostium, and burrowing of pus to distant parts, as also the destruction of tendons and consequent loss of the functions of the limb.

*Case* 9.—Periostitis of leg in a man 30 years old. Has a thick coat of scabs on it from the application of the Iod. of Potass. ointment, which sometimes acts as an irritant.

### DR. VANBUREN'S CLINIQUE.

*Case* 10.—Two cases of coxalgia—hip-joint disease—white swelling—in children 3 and 5 years of age. Is of scrofulous origin. The symptoms present in these cases are: Shortening of limb; swelling and deformity round joint; curvature of the abdominal spine forward; when limb is moved, the motion, instead of being in joint, is in spinal column; head of bone sometimes dislocated—sometimes melted down by disease.

One of these cases has suppurative sinuses in upper part of thigh, and an abscess, which has matter in it now, on the outside of thigh. It is not best to open these abscesses in the neighborhood of diseased hip-joint, if you can avoid it. Promote absorption of matter, if possible; if not, and you find the pus about to give exit to itself, then open—a nice, clean opening with a knife will heal more readily than the rough, jagged one of spontaneous production. *Treatment*—Good diet, good air, iron, cod-liver oil, &c., &c. Nature effects a cure sometimes.

*Case* 11.—Follicular pharyngitis in a young man—a disease that is well defined and well understood, brought into prominent notoriety by Dr. Horace Green, of this city. It consists of inflammation of the mucous follicles of the pharynx, and is confined to that region chiefly; sometimes, not often, the disease gets into the larynx and æsophagus. Dr. Green contends that there is some connection between this disease and phthisis pulmonalis—that it will run into or produce it. This is not so; there is no necessary connection between them. Had just received a monograph from an eminent friend of his in Paris on this subject, in which this connection is denied and refuted.* The disease exists chiefly in clergymen, lawyers, singers, and all persons who use the voice much in public speaking. Treatment is local and general; very difficult of cure. Dissolve 60 grains of chrystalized nitrate of silver in one ounce of glycerine; apply freely, or paint the diseased surface once in three, four or five days. This is better than the watery solution; you can apply it more evenly; the watery solution will stand in drops on the

* This is emphatically a "vexed question" in the great metropolis. The writer is inclined to believe that the members of the profession there are not in a position to judge impartially on the subject, and that their investigations of it, hitherto, have failed to place it in a position satisfactory to the profession at large.

diseased surface the same as an oiled surface. This local treatment alone will not cure the disease; the constitutional treatment is more important—such as sulphur fumes, sulphur baths, pure air, desistance from public speaking, &c., &c.

### DR. PEASLEE ON DISEASES OF CHILDBED.

In case a woman has a dead child, or loses it soon after birth, don't have the milk drawn, if you can help it. Use camphorated oil to soften the breast, and paint a solution of Extract of Belladona on it to dry up the milk. If the breast gets hard and cakey, then have the milk drawn to prevent abscess. Fennel and anise seed, milk punch, &c., will increase the secretion of milk. Retraction of nipple will be remedied by applying to the breast a silver plate having a hole in the centre to admit the nipple. The pressure on the breast will cause the absorption of the adipose matter which projects out over the nipple. Hence, the wearing of cotton pads over the mammæ, or the pressure of corsets or whalebone, will promote the absorption of those glands. He would not allow a daughter of his to wear pads; there was a hollow kind of apparatus, which fitted over the breast, that answered the same purpose as the pads, and was not so objectionable.

### DR. CARNOCHARIS' CLINIQUE.

*Case* 12.—Spina bifida in a child three months old. Hard to do anything with—indeed they are incurable; caused by arrest of development in spinal column. Breschett's doctrine is correct, that the fœtus is developed in distinct halves, which is proved by these arrests of development, occurring in the mesial line. This child looks well otherwise. Exhibited a drawing of another case he had seen four or five years ago, in a child about two years old. It would seem that the tumors might be obliterated by passing ligatures through their base; but it is found that inflammation of the membranes follows, which extends to the brain and proves fatal. The cauda equina is spread out over these tumors.

*Case* 13.—Necrosis of angle of lower jaw, supposed to be caused by the extraction of a tooth. A large piece of bone was extracted, which had an exceedingly offensive odor, the odor of necrosed bone and carcinoma uteri is characteristic.

*Case* 14.—Extensive scrofulous disease of vertebræ in a child two years old. When it occurs so extensively in a child as young as this, it is usually of syphilitic origin, which proves to be so in this case.

*Case* 15.—Almost complete blindness in a woman, forty years old, from iritis. This woman has been blind several years; was struck on the forehead by her husband, (an insane man,) and has had a great deal of affliction, which agencies combined have probably produced it. Lymph is thrown out by the iris, by which it is rendered fixed and immovable, and thus the rays of light are excluded. It is an inflammation that requires active interference.

*Case* 16.—A small steatomatous tumor near angle of jaw in a young man—Contents cherry looking. If the sac is not removed, it will secrete and fill again. Make your incision in the direction of, and not across the vessels and nerves. If you cut across, you would cut the portio dura in this case, and produce some amount of paralysis of the facial muscles for sometime. A case of chronic corneitis, attended with pain in the temple, was treated with an ointment composed of unguent, hydr. extr. bellad. and lard.

Bellevue Hospital.—Service of Dr. McCready.

*Case* 17.—Paraplegia in young man 26 years old. This patient could not walk three months ago; can do so now, to some extent; has been on minute doses of mercury, with which the mouth has been kept *very slightly* sore for most of the time.

When given in this way, no unpleasant effects ever result from it. You can keep the mouth slightly tender in this way for a long time, and the patient eats and drinks, and suffers no inconvenience. The dose is 1-6 to 1-24 of a grain every half hour, hour, or two hours, until the gums are tender, and then often enough to keep them so. We have made great improvement in the use of mercury in the last few years; do not use it in such profusion, but thinks we are going into the other extreme of not using it enough. Given in these minute doses, it does good in many chronic affections, such as iritis, nervous deafness, &c., &c. If this case of paraplegia does not improve any more on the use of it, we will give him strychnia.

*Case* 18.—Scurvy. He had seen but few cases of scurvy until the failure of the potato crop in 1848. Since that time it has prevailed more or less extensively. It was a disease of frequent occurrence before the introduction of the potato into general use. It occurs in persons who live exclvsively on salt meat; the salt impoverishes the meat by absorbing certain of its nutritious constituents necessary to the nutrition of the body, and hence persons fed on those meats have their blood deteriorated and general health impaired. The gums are soft and spongy, and lie loose from the teeth; they bleed easily; there are dark spots on the body, which look like ecchymoses. The eonstituents of meat destroyed by the salt exist to a large extent in the potato and many other vegetables; hence, the raw or boiled potato is one of the remedies for scurvy—the raw is, perhaps, better. Lemonade and citric acid are also good.

*Case* 19.—Albuminaria with delirium tremens. Beware how you give opium in the latter disease when so complicated. There is great tendency to coma in former, which is increased by opium.

### A Rich Day in Pathology.

Dr. Alonzo Clark presented at his clinic two specimens of perforation of the intestine, obtained from patients recently seen by him. It was rather remarkable that two cases somewhat similar should occur in his practice within a few days; and another remarkable feature connected with them was, that the difficulty was diagnosed in both cases. The first case was that of a gentleman who, a short time ago, had returned from the South sick of a fever, which, in the opinion of the attending physician, presented the characters of remittent fever. The case went on for three weeks, with no striking manifestations, and was treated with mild remedies; but little purgative medicine was given. All of a sudden, he was seized with violent pain in the bowels, and it was at this stage of the case that Dr. Clark saw him. His pain was excruciating; abdomen tender and tympanitic; pulse feeble and frequent.

Dr. Clark pronounced it perforation of the intestine, in which opinion the attending physician did not coincide, in consequence of not having seen any of the symptoms usually denominated typhoid in the case. He was put upon large doses of opium—the only remedy from which anything can be expected, under such circumstances—to keep the bowels in absolute quiescence. He died in less than 24 hours after symptoms of perforation had set in.

The case is important, as illustrating the fact that typhoid fever does not always declare itself by its characteristic symptoms, and that this fatal complication may occur in a case comparatively mild in its character. Besides the perforation, there were a few typhoid fever ulcerations also. There was no diarrhœa, and had purgatives been used, they would have been blamed for the occurrence of this lesion.

In the other case, the perforation was in the appendix vermiformis. The gentleman had been in delicate health for some months, and had occasional paroxysms of pain in the bowels, which, however, did not prevent him from attending to his business. A few days ago, he was seized with violent pain in the bowels, somewhat similar to the other case, and Dr. C., on seeing him, pronounced it perforation of the intestine, which it proved to be, from ulceration of the appendix vermiformis.

At the Dead House of Bellevue there were two post-mortems held. One of a boy, 14 years old, who died of effusion on the brain, the effect of blows on the head. There was no fracture of the skull. The other case was that of a large, fat woman, who was brought into the Hospital in a condition approximating that of delirium tremens. She had been beaten by the keeper of a liquor store, and had several contusions—some on her body and some on her head; had also dislocation at the elbow joint from a fall. Chloroform was administered to her, for the purpose of reducing the dislocation, and she died under its influence. The autopsy revealed effusion of serum on the surface of the brain; part of one lung was intensely congested—almost black. Most of the other organs were in a state of fatty degeneration usually found in persons who have long indulged in intoxicating drinks to excess.

Witnessed two post-mortems of children at the Children's Hospital—one four or five months old, died of syphilitic phthisis—a perfect skeleton; its skin during life was of a bluish purple tint; miliary tubercles existed in the lungs; had also intercurrent pneumonia. The other had fatty degeneration of the liver, and enlargement of the mesenteric glands. It was six months old; had an occasional diarrhœa, and died in a convulsion.

